# Study of prevalence of depression in adolescent students of a public school

**DOI:** 10.4103/0972-6748.57859

**Published:** 2009

**Authors:** Vivek Bansal, Sunil Goyal, Kalpana Srivastava

**Affiliations:** Armed Forces Medical College, Pune 411 040, India

**Keywords:** Depression, Students, Public school

## Abstract

**Background:**

Three to nine per cent of teenagers meet the criteria for depression at any one time, and at the end of adolescence, as many as 20% of teenagers report a lifetime prevalence of depression. Usual care by primary care physicians fails to recognize 30-50% of depressed patients.

**Materials and Methods:**

Cross-sectional one-time observational study using simple screening instruments for detecting early symptoms of depression in adolescents. Two psychological instruments were used: GHQ-12 and BDI. Also socio-demographic data (e.g. academic performance, marital harmony of parents, bullying in school, etc) was collected in a separate semi-structured performa. Statistical analysis was done with Fisher’s Exact Test using SPSS17.

**Results:**

15.2% of school-going adolescents were found to be having evidence of distress (GHQ-12 score e14); 18.4% were depressed (BDI score e12); 5.6% students were detected to have positive scores on both the instruments. Certain factors like parental fights, beating at home and inability to cope up with studies were found to be significantly (*P* < 0.05) associated with higher GHQ-12 scores, indicating evidence of distress. Economic difficulty, physical punishment at school, teasing at school and parental fights were significantly (*P* < 0.05) associated with higher BDI scores, indicating depression.

**Conclusion:**

The study highlights the common but ignored problem of depression in adolescence. We recommend that teachers and parents be made aware of this problem with the help of school counselors so that the depressed adolescent can be identified and helped rather than suffer silently.

Just 40 years ago, many physicians doubted the existence of significant depressive disorders in children. However, a growing body of evidence has confirmed that children and adolescents not only experience the whole spectrum of mood disorders but also suffer from the significant morbidity and mortality associated with them.

Despite the high prevalence and substantial impact of depression, detection and treatment in the primary care setting have been suboptimal. Studies have shown that usual care by primary care physicians fails to recognize 30-50% of depressed patients (Simon and Vonkorff, 1995). Because patients in whom depression goes unrecognized cannot be appropriately treated, systematic screening has been advocated as a means of improving detection, treatment, and outcomes of depression.

While improved pediatric diagnosis alone is unlikely to significantly change patient outcomes, recognizing teenagers with depression is the first step to improved depression management. It affects 2% of pre-pubertal children and 5-8% of adolescents. The clinical spectrum of the disease can range from simple sadness to a major depressive or bipolar disorder (Son And Kirchner, 2000). Studies have found that 3-9% of teenagers meet criteria for depression at any one time, and at the end of adolescence, as many as 20% of teenagers report a lifetime prevalence of depression (Zuckerbrotand Jensen, 2006).

Childhood depression, like the depression of adults, can encompass a spectrum of symptoms ranging from normal responses of sadness and disappointment in stressful life events to severe impairment caused by clinical depression that may or may not include evidence of mania (Wolraich *et al*. 1996, Kovacs *et al*. 1994, Weller *et al*. 1996).

Adolescent depression may affect the teen’s socialization, family relations, and performance at school, often with potentially serious long-term consequences. Adolescents with depression are at risk for increased hospitalizations, recurrent depressions, psychosocial impairment, alcohol abuse, and antisocial behaviors as they grow up. Of course, the most devastating outcome of concern for adolescent depression is suicide, the third leading cause of death among older adolescents (Centre for Diseases Control, WISQARS).

Corelational and longitudinal studies have shown that depression is associated with higher rates of smoking, alcohol abuse, unhealthy eating, and infrequent exercise (Haarasilta *et al*., 2004, Franko *et al*. 2005).

No perfect depression screening/assessment tool exists, but a number of adolescent depression assessment instruments do possess adequate psychometric properties to commend their use in depression detection and assessment. Optimal diagnostic procedures should combine the use of depression-specific screening tools as diagnostic aids buttressed by follow-up clinical interviews in which one obtains information from other informants (e.g., parents) and reconciles discrepant information to arrive at an accurate diagnosis and impairment assessment before treatment (Laasa *et al*. 2000).

## MATERIALS AND METHODS

### Study design

It is a cross-sectional one-time observational study using simple screening instruments for detecting early symptoms of depression in adolescents.

Adolescents studying in a public school constituted the study material. All the 125 students studying in 9th standard of the school were evaluated so as to eliminate any selection bias. Questionnaires were given in the class and students were instructed how to fill them in English or Hindi language.

Students were instructed not to write their names to maintain confidentiality. Written consent was taken from everyone and they were explained about the study project.

### Inclusion criteria

1. Adolescents studying in 9^th^ standard of the school.
2. All were overtly healthy.

### Exclusion criteria

1. All students suffering from any kind of chronic disease requiring prescribed medication.
2. All students who had taken any such screening tests before.
3. Any past history of diagnosed mental illness.

The following two instruments were administered:

1. GHQ 12 (General Health Questionairre-12)
2. BDI (Becks Depression Inventory)

The General Health Questionnaire (GHQ) is a subjective measure of psychological wellbeing and Stress Measurement. It has been used in a variety of studies to represent the stress response.

We have used Likert method of scoring in our study. Score of 14 and above is taken as evidence of distress (Goldberg and Williams 1991).

The Beck Depression Inventory (BDI) is a series of questions developed to measure the intensity, severity, and depth of depression in patients with psychiatric diagnoses. The sum of all BDI item scores indicates the severity of depression. Score of 12 and above is taken as Depression. Predictive value of the selected cut-off point, 100% sensitivity, 99% specificity, 0.72 PPV, 1 NPV, and 98% overall diagnostic value (Laasa *et al*. 2000).

Also socio-demographic data (e.g. academic performance, marital harmony of parents, bullying in school, etc) was collected in a separate semi-structured performa.

Statistical analysis was done with Fisher’s Exact Test using SPSS 17.

## RESULTS

In GHQ-12, out of 125 adolescents, 106 did not had any evidence of stress (score <14) and 19(15.2%) were found to be having evidence of distress (score e"14). In BDI, out of 125, 102 were not depressed (score<12) and 23 (18.4%) were depressed (scoree"12).

There were in all 35 students who were detected to have positive scores either in GHQ-12 or BDI. There were seven students who had positive scores on GHQ and BDI [Tables 1 and 2].

**Table 1:** Psychosocial correlates of distress (identified by GHQ) in the adolescents

Factor	Evidence of distress (n=19)		Normal (n=106)		*P* value
	Yes	No	Yes	No	
Parental fights	2	17	0	105	0.022
Economic difficulty	1	18	5	101	1
Loss of parent	0	19	3	103	1
Beating at home	5	14	10	96	0.05
Mental tension due to their parents’ expectations	17	2	84	22	0.526
Happy family	17	2	104	2	0.109
Bullied in school	2	17	9	97	0.674
Physical punishment	4	15	17	89	0.525
Teased at school	4	15	22	84	1
Unable to cope with studies	10	9	24	82	0.01
Have friends	19	0	103	3	1

**Table 2:** Psychosocial correlates of depression (identified by BDI) in the adolescents

Factor	Distressed (n=23)		Normal (n=102)		*P* value
	Yes	No	Yes	No	
Parental fights	2	21	0	102	0.03
Economic difficulty	4	19	2	100	0.01
Loss of parent	1	22	2	100	0.46
Beating at home	5	18	10	92	0.15
Mental tension due to their parents’ expectations	18	5	83	19	0.771
Happy family	22	1	99	3	0.562
Bullied in school	2	21	9	93	1
Physical punishment	9	14	12	90	0.004
Teased at school	9	14	17	85	0.024
Unable to cope with studies	8	15	26	76	0.437
Have friends	23	0	99	3	1

## DISCUSSION

The present study found that 15.2% of the adolescents had evidence of distress and 18.4% were found to be depressed. We tried to find the factors responsible and association of the same with the prevalent stress. We found certain factors like parental fights, beating at home and inability to cope up with studies, to be significantly (*P* <0.05) associated with higher GHQ-12 scores indicating evidence of distress.

Economic difficulty, physical punishment at school, teasing at school and parental fights were significantly (*P* <0.05) associated with higher BDI scores indicating depression.

Factors like bullying in school and parental expectations also are responsible to adding to the stress of an adolescent though it did not reach a statistically significant level in the present study.

The generalizability of the current results is limited since the timing of the study was when the students had just entered 9^th^ standard and were in a jovial mood with comparatively lesser study load but this problem is unavoidable unless multiple studies are done at different times of the year and averaged out.

Cut-off score for BDI ranges from 10-12 depending upon different studies. We took the cut-off score for BDI as 12 thereby increasing the specificity to 99%.

Also, other class students and other schools should also be included in the study for increased generalizability. More extensive studies are required with greater diversity of students, schools and done at different times of the year.

In spite of the limitations, this study points towards the issue of prevalence of depression in adolescence and the purpose of the study is well served to highlight the common but ignored problem.

We recommend that teachers and parents be made aware of this problem with the help of school counselors so that the depressed adolescent can be identified and helped rather than suffer silently.
